# Lyssavirus in Japanese Pipistrelle, Taiwan

**DOI:** 10.3201/eid2404.171696

**Published:** 2018-04

**Authors:** Shu-Chia Hu, Chao-Lung Hsu, Ming-Shiuh Lee, Yang-Chang Tu, Jen-Chieh Chang, Chieh-Hao Wu, Shu-Hwae Lee, Lu-Jen Ting, Kwok-Rong Tsai, Ming-Chu Cheng, Wen-Jane Tu, Wei-Cheng Hsu

**Affiliations:** Animal Health Research Institute, New Taipei City, Taiwan (S.-C. Hu, M.-S. Lee, Y.-C. Tu, J.-C. Chang, C.-H. Wu, L.-J. Ting, K.-R. Tsai, W.-J. Tu, W.-C. Hsu);; National Taiwan University, Taipei City, Taiwan (C.-L. Hsu);; Bat Conservation Society of Taipei, Taipei City (C.-L. Hsu);; Animal Health Research Institute, Miaoli County, Taiwan (S.-H. Lee);; National Pingtung University of Science and Technology, Neipu Township, Pingtung County, Taiwan (M.-C. Cheng)

**Keywords:** lyssavirus, bat, Japanese pipistrelle, Pipistrellus abramus, Taiwan, viruses

## Abstract

A putative new lyssavirus was found in 2 Japanese pipistrelles (*Pipistrellus abramus*) in Taiwan in 2016 and 2017. The concatenated coding regions of the virus showed 62.9%–75.1% nucleotide identities to the other 16 species of lyssavirus, suggesting that it may be representative of a new species of this virus.

The *Lyssavirus* genus within the family *Rhabdoviridae* is composed of 14 species of lyssavirus: rabies lyssavirus (RABV), Lagos bat lyssavirus (LBV), Mokola lyssavirus (MOKV), Duvenhage lyssavirus (DUVV), European bat 1 lyssavirus (EBLV-1), European bat 2 lyssavirus (EBLV-2), Australian bat lyssavirus (ABLV), Aravan lyssavirus (ARAV), Khujand lyssavirus (KHUV), Irkut lyssavirus (IRKV), Shimoni bat lyssavirus (SHIBV), Bokeloh bat lyssavirus (BBLV), West Caucasian bat lyssavirus (WCBV), and Ikoma lyssavirus (IKOV) (*1*). In addition, Lleida bat lyssavirus (LLEBV) (*1**,**2*) and Gannoruwa bat lyssavirus (GBLV) (*3*) were recently identified in bats, but their taxonomic statuses have not been determined by the International Committee on the Taxonomy of Viruses. The genus *Lyssavirus* can be subdivided into phylogroup 1 (RABV, DUVV, EBLV-1, EBLV-2, ABLV, ARAV, KHUV, IRKV, BBLV, and GBLV) and phylogroup 2 (LBV, MOKV, and SHIBV) according to genetic distances and serologic cross-reactivity (*1**–**3*). The remaining species, WCBV, IKOV, and LLEBV, cannot be included in either of these phylogroups (*1**,**2*).

Bats are the natural hosts of most lyssaviruses, with the exceptions of MOKV and IKOV, which have not been identified in any bats (*1**–**4*). Information about lyssaviruses in bats in Asia is limited. In Central Asia, ARAV was identified in the lesser mouse-eared bat (*Myotis blythi*) in Kyrgyzstan in 1991, and KHUV was identified in the whiskered bat (*M. mystacinus*) in Tajikistan in 2001 (*5*). In South Asia, GBLV was identified in the Indian flying fox (*Pteropus medius*) in Sri Lanka in 2015 (*3*). Although IRKV was identified in the greater tube-nosed bat (*Murina leucogaster*) in China in 2012 (*6*), knowledge of the exact species and locations of lyssaviruses in East Asia bat populations remains limited.

In this article, we report a putative new lyssavirus isolated during our surveillance program in Taiwan. Our discovery suggests that this lyssavirus may be representative of a new species, based on genetic distance.

## The Study

Specimens for this study were collected under a permit issued by the Forestry Bureau, Council of Agriculture, Executive Yuan, Taiwan (document no. 1055104969). From 2014 through the end of May 2017, a total of 332 bat carcasses from 13 species were collected for lyssavirus surveillance. Of the collected individuals, 2 tested positive for the virus by direct fluorescent antibody testing and reverse transcription PCR (*7**–**9*). The first bat showing loss of appetite without specific clinical signs was found in Tainan City and died on July 2, 2016. The second bat was found dead in Yunlin County on April 12, 2017, and the carcass was shipped to the Animal Health Research Institute (AHRI) in New Taipei City. We obtained two 428-bp amplicons (N113F/N304R, containing the partial nucleoprotein [N] gene and phosphoprotein [P] gene) from these cases using lyssavirus screen primers (Table 1); we then subjected their sequences to BLAST (https://www.ncbi.nlm.nih.gov/BLAST/), querying the GenBank database. Both sequences were similar to lyssaviruses, with nucleotide identities <79%.The 2 bats were identified as Japanese pipistrelle (*Pipistrellus abramus*), or Japanese house bat, by morphology (J.-T. Wu, Taxonomic study of the genus *Pipistrellus* [chiroptera: vespertilionidae] in Taiwan. Master’s thesis, Department of Biological Resources, National Chiayi University, Chiayi City, Taiwan, 2007 [in Chinese]) and DNA barcoding (based on subunit 1 of the mitochondrial protein NADH dehydrogenase [ND1] gene) (*10*). The 2 sequences of partial ND1 genes were then submitted to GenBank; the first had been designated as 2016-2300 (GenBank accession no. MG763889) and the second 2017-1502 (GenBank accession no. MG763890).

**Table 1:** Lyssavirus screen primers and the 12 amplifying primer sets used to identify Taiwan bat lyssavirus, a putative new lyssavirus found in 2 Japanese pipistrelles (*Pipistrellus abramus*) in Taiwan in 2016 and 2017

Primer name	Sequence, 5′ → 3′	Position*
Lyssavirus screen		
JW12 (*7*)	ATGTAACACCYCTACAATG	
N165–146 (*7*)	GCAGGGTAYTTRTACTCATA	
N113F (*8*)	GTAGGATGCTATATGGG	
N304R (*9*)	TTGACGAAGATCTTGCTCAT	
Lyssavirus full genome		
TWBLV 1F	ACGCTTAACGACAAAAYC	1–18*
TWBLV 1R	TCTTGCATTTCTTTCTCATC	1154–1173
TWBLV 2F	TTCGTAGGATGTTACATGGG	1010–1029
TWBLV 2R	taaaaatatcccagaagatc	2174–2193
TWBLV 3F	AGARATAGCWCATCAGATWGC	2118–2138
TWBLV 3R	CTATTGTGTGGCACCATWAC	3205–3224
TWBLV 4F	GATGAGGATAAGAACACATC	3072–3091
TWBLV 4R	TCCTGAAGTGACTGAGTTTTC	4274–4294
TWBLV 5F	CTGATGGAYGGRTCATGGGT	4081–4100
TWBLV 5R	GAGACAGGAGCCGGAGTCTT	5280–5299
TWBLV 6F	AACAGGTAGCTCCCGAGTTTGTTC	4881–4904
TWBLV 6R	CTGAGTGAGACCCATGTATCCAAA	5767–5790
TWBLV 7F	ACTGAGGTTTATGATGACCC	5478–5497
TWBLV 7R	CCCCAGTGTCTATARCAWCC	6561–6580
TWBLV 8F	CATTCTTTGGGGGATTTCCC	6426–6445
TWBLV 8R	GTTTGTGATTCTCTRTCWATC	7595–7615
TWBLV 9F	CATGCTGGAACGGTCAGGAYG	7522–7542
TWBLV 9R	CTGAGTTAAAGAAAGATTCTT	8662–8682
TWBLV 10F	CTCAGTGAGTTRTTYAGCTC	8547–8566
TWBLV 10R	CAGATAGAAGAGCCTATT	9746–9763
TWBLV 11F	CATGATTCAGGGTAYAAYGA	9648–9667
TWBLV 11R	GTCTGTAACTTCTGCATCAC	10862–10871
TWBLV 12F	ATCTGGGAAAAGCCATCAGA	10755–10774
TWBLV 12R	ACGCTTAACAAAAAAAACAA	11961–11980

*Compared with reference genomic sequence, Irkut lyssavirus (GenBank accession no. NC020809). TWBLV, Taiwan bat lyssavirus.

We isolated lyssavirus successfully from the 2 bats’ brains; we confirmed the identity of lyssavirus morphologically by electron microscopy (*11*) and molecularly by nucleotide sequencing. In addition, after we isolated the viruses from the brain tissues, we performed reverse transcription PCR and virus isolation for visceral organs and salivary glands; both tests were positive for the salivary glands of both bats.

We determined nucleotide sequences of the genome of the isolated viruses, designated Taiwan bat lyssavirus (TWBLV). The first TWBLV isolate was designated TWBLV/TN/2016 (GenBank accession no. MF472710), and the second TWBLV/YL/2017 (GenBank accession no. MF472709). We amplified the nucleoprotein (N), phosphoprotein (P), matrix protein (M), glycoprotein (G), and RNA-dependent RNA polymerase (L) genes of the 2 TWBLV isolates by 12 primer sets designed by the AHRI (Table 1). The genome length of the 2 TWBLV isolates was 11,988 bases, with G+C contents of 43.66% for TWBLV/TN/2016 and 43.83% for TWBLV/YL/2017. The genomic organization was similar to those of other lyssaviruses: 3′ untranslated region (UTR), 70 nt; N, 1356 nt; N-P UTR, 99 nt; P, 897 nt; P-M UTR, 82 nt; M, 609 nt; M-G UTR, 212 nt; G,1629 nt; G-L UTR, 518 nt; L, 6384 nt; and 5′ UTR, 132 nt. The 2 TWBLV isolates showed 98.7% nucleotide identity in the N gene and 98.6% nucleotide identity in the concatenated coding genes (N+P+M+G+L). Nucleotide identities of different genes between the TWBLVs and the other 16 lyssaviruses are listed in Table 2. For the N gene, the TWBLVs had the highest identities with IRKV (79.0%–80.6%), followed by EBLV-1 (78.8%–79.4%). The TWBLVs shared nucleotide identities with the concatenated coding genes of EBLV-1 (75.1%), followed by IRKV (74.0%). The phylogenetic analysis demonstrated that lyssaviruses were separated into 2 phylogroups; the TWBLVs were grouped into phylogroup 1 and clustered with the EBLV-1, IRKV, and DUVV (Figure).

**Table 2:** Nucleotide identities for the N, P, M, G, L genes and the concatenated coding genes between Taiwan bat lyssavirus, a putative new lyssavirus found in 2 Japanese pipistrelles (*Pipistrellus abramus*) in Taiwan in 2016 and 2017, compared with other lyssaviruses*

Lyssavirus species (GenBank accession no.)						Concatenated coding genes (N+P+M+G+L)
	Identity, %					
	N	P	M	G	L	
Rabies lyssavirus (NC001542)	75.2	62.8–63.1	74.6–74.8	61.6–62.3	71.7	70.0–70.1
Lagos bat lyssavirus (NC020807)	73.5–73.7	53.8–54.1	73.6	55.0–55.6	71.3–71.4	67.8–67.9
Mokola lyssavirus (NC006429)	72.7–73.3	52.2–52.4	72.0–72.6	56.7–56.9	70.6–70.7	67.3 −67.4
Duvenhage lyssavirus (NC020810)	77.7–77.9	67.5–67.6	78.4–79.2	63.9–64.0	74.8–74.9	73.2
European bat 1 lyssavirus (NC009527)	78.8–79.4	69.6–69.7	80.9–81.1	66.1	76.7–76.8	75.1
European bat 2 lyssavirus (NC009528)	75.3–75.6	66.0–66.3	79.2–79.5	64.5–64.6	73.7	72.2–72.3
Australian bat lyssavirus (NC003243)	76.3–76.5	62.2	72.1	61.7–62.0	72.3–72.5	70.4
Aravan lyssavirus (NC020808)	76.5 −76.7	65.6–66	80.3–80.6	64.4–64.5	74.6–74.9	72.9–73.1
Khujand lyssavirus (NC025385)	74.6–74.7	67.0–67.1	78.6–78.7	64.5–64.9	74.2–74.3	72.4–72.5
Irkut lyssavirus (NC020809)	79.0–79.1	68.3–68.6	80.3	65.3–65.8	75.3–75.4	74
Irkut lyssavirus-THChina12 (JX442979)	80.5–80.6	68.7–69.0	79.8–80.0	64–64.6	75.1–75.4	74
West Caucasian bat lyssavirus (NC025377)	72.3–72.7	51.2–51.5	71.4	52.0	68.3–68.4	65–65.2
Shimoni bat lyssavirus (NC025365)	74.1–74.5	54.9–55.1	74.2–74.6	56.2–56.4	71.1–71.2	68.2
Ikoma lyssavirus (NC018629)	69.7–69.8	51.9–52.3	69.3–69.6	50.9–51.2	65.4–65.6	62.9
Bokeloh bat lyssavirus (NC025251)	75.0–75.2	66.0–66.3	78.2	64.4–64.5	74.1–74.2	72.3–72.4
Lleida bat lyssavirus (NC031955)	69.2–69.5	50.8–50.9	70.4–70.9	51.2	66.2	63.2–63.3
Gannoruwa bat lyssavirus (NC031988)	75.5–75.7	62.8–63.1	75.4–75.7	63.7–64.3	73.5–73.6	71.5–71.6
*G, glycoprotein; L, RNA-dependent RNA polymerase; M, matrix protein; N, nucleoprotein; P, phosphoprotein.						

**Figure:**
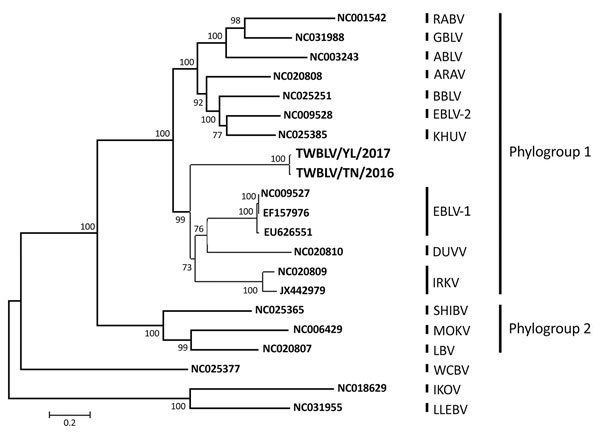
Phylogenetic relationship of TWBLV (boldface), a putative new lyssavirus found in 2 Japanese pipistrelles (*Pipistrellus abramus*) in Taiwan in 2016 and 2017, compared with other lyssaviruses. Using the concatenated coding genes, we constructed the phylogenetic tree by using the maximum-likelihood method with the general time reversible plus invariant sites plus gamma 4 model. Numbers at the nodes indicate bootstrap confidence values (1,000 replicates) for the groups being composed of virus genes at the right of the nodes. GenBank accession numbers are provided for reference viruses. Scale bar indicates nucleotide substitutions per site. ABLV, Australia bat lyssavirus; ARAV, Aravan lyssavirus; BBLV, Bokeloh bat lyssavirus; DUVV, Duvenhage lyssavirus; EBLV-1, European bat lyssavirus type 1; EBLV-2, European bat lyssavirus type 2; IKOV, Ikoma lyssavirus; IRKV, Irkut lyssavirus; KHUV, Khujand lyssavirus; LBV, Lagos bat lyssavirus; LLEBV, Lleida bat lyssavirus; MOKV, Mokola lyssavirus; SHIBV, Shimoni bat lyssavirus; RABV, rabies lyssavirus; TWBLV, Taiwan bat lyssavirus; WCBV, West Caucasian bat lyssavirus.

## Conclusions

We report a lyssavirus, TWBLV, that is closely related to EBLV-1, IRKV, and DUVV. The full-length nucleotide sequence of the concatenated coding genes of TWBLV showed 62.9%–75.1% nucleotide identities to the other 16 lyssaviruses (Table 2), and TWBLV was the most closely related to EBLV-1. The demarcation criteria of lyssavirus species, established by the International Committee on the Taxonomy of Viruses, include genetic distance, topology, antigenic patterns, and additional characteristics (*12*). Based on genetic distance, no similarity to the other lyssavirus species of more than 75.1% nucleotide identities of the concatenated coding genes of TWBLVs suggested that the isolated TWBLV was a new lyssavirus species.

The presence of TWBLV in the bats’ salivary glands suggested that TWBLV may be shed through saliva. The study showed that the bat in East Asia could be infected with lyssavirus; however, because of the limited surveillance, the epidemiology of lyssavirus in Japanese pipistrelle and other bat species is still unclear. This uncertainty is likely to raise a public health concern in countries in Asia.

In conclusion, a lyssavirus, TWBLV, was identified in Japanese pipistrelle, and the infected bats may shed the virus through saliva. Japanese pipistrelle is a common insectivorous bat of low-altitude urban areas in East Asia (*13**,**14**)*. Persons in countries in Asia should be aware to seek proper prophylaxis immediately if bitten by a bat. Studies on the epidemiology and pathogenicity of TWBLV are necessary to further characterize the virus.
